# Integrating Veterinary Diagnostic Laboratories for Emergency Use Testing during Pandemics^1^

**DOI:** 10.3201/eid3002.230562

**Published:** 2024-02

**Authors:** Natasha F. Hodges, McKenzie Sparrer, Tyler Sherman, Treana Mayer, Danielle R. Adney, Izabela Ragan, Molly Carpenter, Christie Mayo, Tracy L. Webb

**Affiliations:** Colorado State University, Fort Collins, Colorado, USA (N.F. Hodges, M. Sparrer, T. Sherman, T. Mayer, I. Ragan, M. Carpenter, C. Mayo, T.L. Webb);; Lovelace Biomedical, Albuquerque, New Mexico, USA (D.R. Adney)

**Keywords:** bioterrorism and preparedness, SARS-CoV-2, coronaviruses, viruses, COVID-19, emergency use testing, veterinary diagnostic laboratories, veterinary diagnostic services, disease outbreaks, respiratory infections, One Health, pandemics, United States

## Abstract

The SARS-CoV-2 pandemic showed limitations in human outbreak testing. Veterinary diagnostic laboratories (VDLs) possess capabilities to bolster emergency test capacity. Surveys from 26 participating VDLs found human SARS-CoV-2 testing was mutually beneficial, including One Health benefits. VDLs indicated testing >3.8 million human samples during the pandemic, which included some challenges.

After emergence of SARS-CoV-2 in late January 2020, diagnostic testing was fraught with challenges. As cases increased, public health agencies struggled to provide timely support, prompting veterinary diagnostic laboratories (VDLs) to assist with processing human SARS-CoV-2 samples (*1*). VDLs regularly conduct diagnostic testing for infectious agents and maintain the necessary equipment, personnel, facilities, and protocols for animal disease testing. Currently, there are 60 university- or state-affiliated VDLs across the United States (*2*). On April 1, 2020, the World Organization for Animal Health published guidance stating that VDLs possess the resources and personnel expertise to help human diagnostic laboratories meet the demand for SARS-CoV-2 testing (*3**,**4*).

To assess VDL participation in human testing, we distributed a 14-question survey (Appendix) to 52 VDLs across the United States that had available email addresses. The study was reviewed by Colorado State University's Institutional Review Board (Protocol no. 3620), and respondent answers were deidentified before analysis. The first question queried whether human SARS-CoV-2 samples were tested and required an affirmative response to continue the survey. Subsequent questions were optional. Responses were gathered during July 7–December 22, 2022. Two follow-up reminders were sent during the open survey period. Responses were received from 26 (43.3%) of the 60 VDLs overall or 26 (50%) of the 52 VDLs that were contacted. Nine respondents indicated no human testing, and 17 (65.4%) of the 26 responding VDLs reported performing human testing. When >1 response was received from the same VDL (5 VDLs submitted >1 survey), numeric data were averaged, and all free text entries were included.

The duration of human testing across responding VDLs ranged from 5 to 31 months; average testing duration was 20 months (95% CI 15.7–24.4 months). Twelve VDLs reported testing numbers ranging from 6,000 to 200,000 samples/facility (95% CI 67,200–224,000 samples/facility). One additional facility reported 2.1 million samples tested, totaling ≈3.8 million samples. When asked to declare populations served, VDLs indicated staff and students as the largest testing group, followed by local community as the second largest and long-term care facilities as the third largest (Figure).

**Figure:**
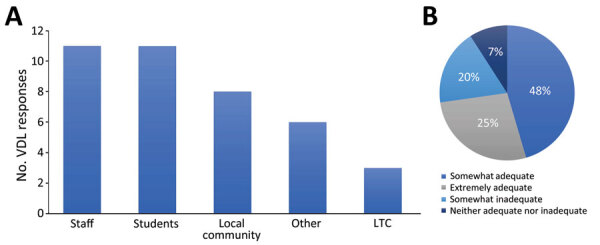
Population served (A) and perceived funding adequacy (B) of veterinary diagnostic laboratories (VDLs) conducting human SARS-CoV-2 testing, United States. A) Sum of responses for each of 5 selectable testing population types as reported by 13 of 17 VDLs performing human SARS-CoV-2 testing that responded to this optional question. VDLs could select any combination of answers that represented their specific testing populations. LTC, long-term care. B) Percentages of the 11 of 17 VDLs performing human SARS-CoV-2 testing that responded to the optional question to select 1 of 5 funding adequacy descriptions (no responses were received for inadequate).

We asked VDLs to rank the main challenges for human SARS-CoV-2 testing by using a rank-order question with 6 predefined and 3 open-response options. The survey asked respondents to rank items from 1 to 9, where 1 represented not challenging and 9 represented most challenging; each rank was selected only once. Personnel was the biggest challenge reported (average rank 7.7). Supplies (5.7) and certification (5.4) were moderately challenging on average, and facilities (3.7), training (3.6), and funding (3.0) were less challenging.

All respondents reported that the experience was beneficial to overall work going forward, 66.7% strongly agreeing and 33.3% somewhat agreeing. When asked to elaborate, 14 respondents included opportunities to optimize personnel, optimizing testing workflows, increased recognition, and relationship building as benefits. Most (83%) surveyed VDLs responded yes to the question of whether their laboratories experienced One Health benefits related to performing human sample testing (i.e., interagency connections, interdisciplinary work, or ideas that came from testing). In a follow-up write-in question, respondents’ comments included improved awareness and recognition, relationship building, resultant collaborative opportunities, and sharing of information.

Two final questions asked about lessons learned. Responses supported planning and readiness with flexible workspaces, tested workflows, available trained personnel, financial needs, quality sample management, and validated equipment. Knowledge about Clinical Laboratory Improvement Amendments regulations and certification (https://dch.georgia.gov/divisionsoffices/hfrd/facilities-provider-information/clia) was mentioned in 40% of responses. Additional comments focused on the need for staff support, challenges to managing sample requirements, balancing multiple disease outbreaks, the need for establishing relationships, and pride in accomplishments.

Challenges reported through the survey included access to supplies as supply chain disruptions contributed to difficulty in procuring instrumentation, laboratory consumables, and personal protective equipment. Challenges related to personnel included availability of staff that met state-level criteria for testing, such as Clinical Laboratory Improvement Amendments certification. Further complications experienced by many VDLs included software integration and maintenance for reporting test results, as well as coordination of sample collection and receiving and handling from collaborating entities. The SARS-CoV-2 caseload was often in addition to existing testing needs, requiring longer or irregular working hours to meet expected turnaround times. For frontline pandemic workers, those conditions might have contributed to accelerated staff burnout and reported staff challenges.

The SARS-CoV-2 pandemic offers a One Health case model, given that both humans and animals may become infected and environmental detection is possible (e.g., wastewater) (*5*,*6*). As recently demonstrated, human testing facilities might struggle to meet emergency public health demands without additional support; however, laboratories that regularly test other zoonotic and nonzoonotic pathogens can help meet testing needs. Many of the responding VDLs reported mutually beneficial outcomes from participating in human SARS-CoV-2 testing, particularly in the form of new interagency relationships, shared information, and improved recognition. Similar coordinated, collaborative efforts might be particularly useful in mitigating future pandemics and improving disease response outcomes (*7*,*8*).

Appendix Additional information on integrating veterinary diagnostic laboratories for emergency use testing during pandemics.
