# Supplementary material for: Integrating Veterinary Diagnostic Laboratories for Emergency Use Testing during Pandemics
Source: Emerg Infect Dis. 2024 Feb;30(2):386–8. doi: 10.3201/eid3002.230562 (PMC10826766; doi:10.3201/eid3002.230562)
Supplement: Appendix — Additional information on integrating veterinary diagnostic laboratories for emergency use testing during pandemics. [file 23-0562-Techapp-s1.pdf]

Article DOI: https://doi.org/10.3201/eid3002.230562

EID cannot ensure accessibility for Supplemental Materials supplied by authors. Readers who have difficulty accessing supplementary content should contact the authors for assistance.

Integrating Veterinary Diagnostic Laboratories for Emergency Use Testing during Pandemics

Appendix

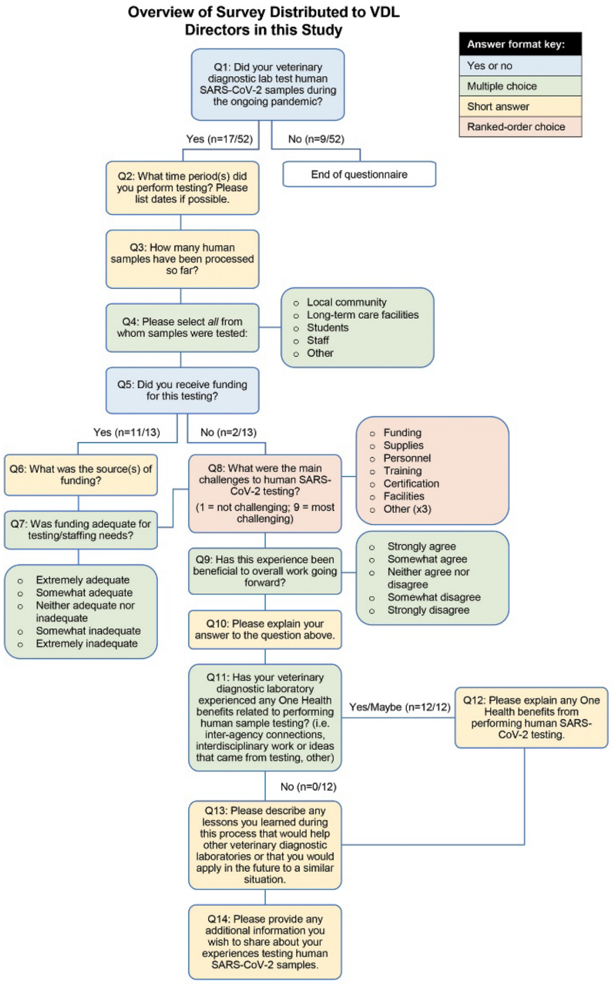

Appendix Figure. Overview of survey distributed to veterinary diagnostic laboratory directors in this study. SARS-CoV-2, severe acute respiratory syndrome coronavirus 2; VDL, veterinary diagnostic laboratories.
